# Metamemory monitoring in Alzheimer’s disease A systematic review

**DOI:** 10.1590/1980-57642018dn12-040002

**Published:** 2018

**Authors:** Michelle Brandt, Raquel Luiza Santos de Carvalho, Tatiana Belfort, Marcia Cristina Nascimento Dourado

**Affiliations:** 1 MS, Center for Alzheimer’s disease and Related Disorders, Institute of Psychiatry, Universidade Federal do Rio de Janeiro, RJ, Brazil; 2 PhD, Center for Alzheimer’s disease and Related Disorders, Institute of Psychiatry, Universidade Federal do Rio de Janeiro, RJ, Brazil.

**Keywords:** metacognition, metamemory, dementia, Alzheimer´s disease, cognition, metacognição, metamemória, demência, doença de Alzheimer, cognição

## Abstract

Metamemory is the awareness of one’s own knowledge and control of memory, and refers to the online ability to gather information about the current state of the memory system.

**Objective:**

Metamemory is one’s own knowledge and control of memory. A systematic review was performed to identify the types of tasks used for evaluating metamemory monitoring, the stimuli used in these tasks, their limitations and the outcomes in people with Alzheimer’s disease (PwAD).

**Methods:**

This systematic review followed PRISMA methodology. A search of Pubmed, Scopus and Web of Science electronic databases was carried out in September, 2018, identifying experimental investigations of metamemory and dementia.

**Results:**

We included 21 studies. The most common tasks used were judgement of learning, feeling of knowing, judgement of confidence and global prediction. The rates of discrepancy between PwAD and caregivers still need further research. The Rey Auditory Verbal Learning Test was the most used list of words. PwAD are able to accurately rate their memory functioning and performance, when the evaluation is done soon afterwards. PwAD tend to overestimate their functioning and performance when the judgement involves forward-looking vision.

**Conclusion:**

In the context of metamemory impairment, clinicians and caregivers should seek interventions aiming to identify compensatory styles of functioning. This systematic review provides initial evidence for the use of metamemory measures as part of broader assessments evaluating Alzheimer’s disease.

## RESUMO

Metamemoria é a consciência do próprio conhecimento e controle da memória, e refere-se à capacidade online de reunir informações sobre o estado atual do sistema de memória.

**Objetivo:**

Metamemória é a consciência sobre o próprio conhecimento e controle da memória. Nós conduzimos uma revisão sistemática para identificar os tipos de tarefa usadas para avaliar o monitoramento da metamemória, os estímulos usados nessas tarefas, suas limitações e resultados em pessoas com doenças de Alzheimer.

**Métodos:**

Esta revisão sistemática usou a metodologia PRISMA. Uma busca nas bases Pubmed, Scopus e Web of Science foi feita em Setembro de 2018. Foram identificados estudos experimentais em metamória e demência.

**Resultados:**

Foram incluídos 21 estudos que se enquadravam nos critérios de inclusão. As tarefas mais comuns foram “judgement of learning”, “feeling of knowing”, “judgement of confidence” and “global prediction”. As discrepâncias, em termos de monitoramento de metamemória, ainda necessitam de pesquisas futuras. O Rey Auditory Verbal Learning Test foi a lista de palavras mais usada. Pessoas com doença de Alzheimer são capazes de avaliar de forma acurada o seu funcionamento da memória, quando a avaliação é feita em um momento posterior. Eles tendem a superestimar seu funcionamento quando o julgamento é feito em uma visão de futuro.

**Conclusão:**

No contexto do comprometimento da metamemória, é necessário que clínicos e cuidadores procurem intervenções com o objetivo de identificar estilos compensatórios de funcionamento. Assim, esta revisão sistemática fornece evidências iniciais sobre o uso de medidas de metamemória como parte de avaliações mais amplas na doença de Alzheimer.

Metamemory is the awareness of one’s own knowledge and control of memory, and refers to the online ability to gather information about the current state of the memory system.^1^ It is a higher order cognitive process that involves memory function, beliefs, attitudes, sensations and knowledge about memory function.^2^ The ability to accurately monitor our own cognitive abilities is critical to our effective functioning.^3^

The metamemory concept is a specific term from a global construct, namely, metacognition. Metacognition is the capacity of people to know about their own perceptions, memories, decisions, and actions. It refers to realistic perception of one’s situation, functioning or performance and the resulting implications, which may be expressed explicitly or implicitly.^4^ Accurate metacognition enables people to accurately assess how good are one’s learning, cognition, or memory, for example, both in general and for particular items that will and will not be performed correctly.^4^

Various metacognitive models have been proposed to explain how individuals maintain or lose awareness of their cognitive functioning. One popular model is the Cognitive Awareness Model (CAM).^5^ This model provides a neurocognitive explanation of unawareness, acknowledging the heterogeneous bases of awareness deficits.^4^ The CAM attempts to account for deficits at different stages of information processing that result in a particular type of awareness error and when these errors are either undetected or not perceived as affectively salient. The importance of emotional dysregulation in unawareness errors requires further investigation for a better comprehension of affective signature to motivate self-monitoring.^4^

In the CAM model,^5^ a mnemonic anosognosia can occur when there is a failure to update one’s autobiographical knowledge regarding cognitive abilities in light of cognitive failures.^4^ Thus, the individual retains an outdated representation of the self’s ability. Morris and Mograbi (2013)^5^ suggest a distinction between explicit and implicit information processing, leading to a potential dissociation between the conscious versus unconscious monitoring of cognitive failures. This breakdown in the integration between explicit and implicit systems may contribute to the failure to exhibit explicit awareness of such errors. The individual may show preserved implicit monitoring wherein the person adjusts or adapts his or her everyday functioning to accommodate cognitive deficiencies, or demonstrates emotional reactions that suggest implicit monitoring of cognitive failures.^5^

There are two main processes related to metamemory: monitoring and control.^1^ Monitoring is the mechanism by which individuals evaluate the accuracy of potential responses. It is based on a collection of information about one’s own knowledge and memory performance.^6^ Control refers to the self-regulation processes of one’s own memory behavior.^7^ The two processes operate in a feedback loop, that is, there is a strong link between both concepts and we can control our memory function through memory monitoring.^7^ For example, self-monitoring abilities support activities of daily living in a way that simple tasks, such as remembering to take medication, recruit the capacity of accessing memory for problem solving.^8^

People with Alzheimer disease (PwAD) frequently have anosognosia, i.e. they are unaware of the disease or fail to appreciate the degree to which their disorder impacts their functioning.^9^ Studies show that, besides anosognosia, PwAD tend to present decline in metacognitive processes.^3^^,^ 10 ^,^ 11 Anosognosia is one hypothesis for metamemory deficits in PwAD.^7^ Therefore, the constructs and measures of metamemory can be used to evaluate, and should increase our understanding of, the cognitive process underlying the lack of awareness of memory deficits in Alzheimer’s disease (AD).

Several studies show that, in early stages of dementia, many individuals experience difficulties in awareness of memory loss.^9^^,^ 11 ^-^ 13 These failures have implications in supporting activities of daily living. Conversely, when awareness of deficits is preserved, self-monitoring can lead individuals to engage in compensatory strategies to avoid forgetting.^8^^,^ 14 ^,^ 15 However, studies tend not to investigate the differences of monitoring metamemory tasks and the stimuli used in these tasks in PwAD. This type of research is important to evaluate the influence of tasks and stimuli used for monitoring memory capacity. The present review is aimed at identifying the types of tasks used to evaluate metamemory monitoring, the stimuli used in these tasks, their limitations and the outcomes in PwAD.

## METHODS

This systematic review was conducted according to the Preferred Reporting Items for Systematic Reviews and Meta-Analyses (PRISMA).

Literature searches were carried out in September, 2018 using the following electronic databases: Pubmed, Scopus and Web of Science. The search keywords included: “metacognition and dementia”, “metacognition and Alzheimer’s disease”, “metamemory and dementia”, “metamemory and Alzheimer’s disease”, “judgment of learning and dementia”, judgment of learning and Alzheimer’s disease”, “feeling-of-knowing and dementia”, “feeling-of-knowing and Alzheimer’s disease”, “judgment of confidence and dementia”, “judgment of confidence and Alzheimer’s disease”, “metacognitive Control and dementia”, “metacognitive control and Alzheimer’s disease”, “metacognitive knowledge and dementia”, “metacognitive knowledge and Alzheimer’s disease”, “metacognitive monitoring and dementia”, “metacognitive monitoring and Alzheimer’s disease”.

Our inclusion criteria encompassed studies written in an English language peer-reviewed journal, with experimental design based on a sample of PwAD. The exclusion criteria were: (1) participants with pre-clinical dementia conditions, namely, mild cognitive impairment (MCI), (2) other dementia subtypes or clinical pathologies and psychiatric comorbidities, (3) papers that were not about metamemory, (4) studies not focused on tasks, (5) and opinion papers or reviews. Two authors (RLSC & MCND) independently screened titles and abstracts to identify eligible papers. We excluded all studies that clearly did not meet all inclusion criteria or that met at least one of the exclusion criteria. Afterwards, two authors (MB & MCND) independently reviewed the full publications of the remaining papers and held consensus meetings to discuss any disagreement and reach a consensus on inclusion. When necessary, a third co-author of this paper (TB) clarified study eligibility.

## RESULTS

**Figure 1:**
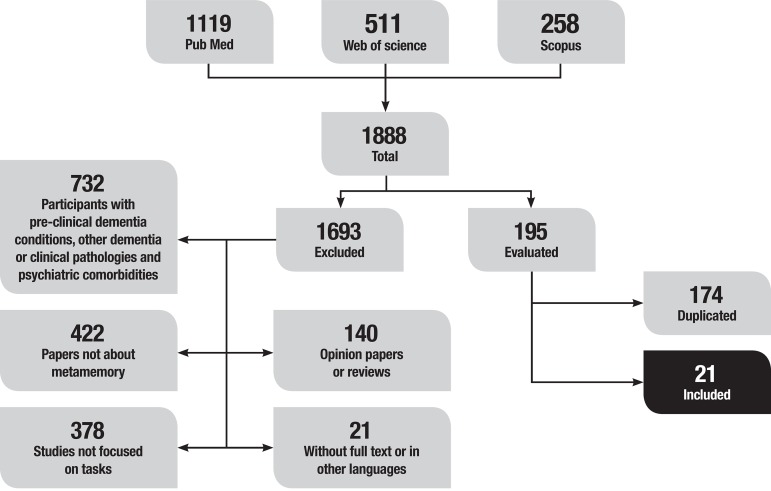
Literature on metamemory flow diagram

Initially, 1888 records were identified through database searching: 1119 on PubMed, 511 on Web of Science and 258 on Scopus. The 195 studies that remained after applying the exclusion criteria were retrieved for potential use and the information of the full-text version of each study was evaluated. Cross-referencing of reference lists of all selected papers was undertaken. After duplicates were removed, the total number of studies was 21. The flow diagram depicting the different phases of the systematic review is shown in Figure 1.

Different tasks were used to evaluate metamemory monitoring: feeling of knowing (FOK), Ease of learning (EOL), Judgment of learning (JOL), Retrospective Confidence Rating (CR), Response to Feedback, Metamemory Accuracy, Recall readiness, Ranking judgment, judgment of confidence (JOC), Global Judgment of performance, Objective Judgment Discrepancy (OJD), Subjective Rating Discrepancy (SRD), The Experimenter Rating Scale (ERS), Objective Judgement (OJ), Subjective Rating of Memory Function, Online and Offline Metamemory.

The selected studies were organized according to the type of task: Feeling of Knowing (FOK) metamemory tasks (Table 1); Judgment of Confidence (JOC) metamemory tasks (Table 2); Judgment of Learning (JOL) metamemory tasks (Table 3), and Global Prediction and other metamemory tasks (Table 4). The most common metamemory monitoring tasks presented in the studies were: the JOL, FOK, JOC and Global Prediction.

**Table 1:** Summary of studys that used Feeling of Knowing (FOK).

Study	Sample	Measures	Tasks	Main results
1. Cosentino et al., 2016	49 - mild to moderate AD (22 - awareness 27 - unaware)	• A modified version of anosognosia rating scale. • A modified episodic FOK task. • PVLT • Executive Functioning • Phonemic fluency test	• Prospective condition - prospective judgments for each item in response to written questions. • Retrospective condition - identical to the prospective condition, with the additional component of a retrospective evaluation after every item, regardless of accuracy. • Feedback condition - identical to the prospective condition, except that the examiner provided feedback regarding recognition memory performance after each item. • Metamemory accuracy - relative accuracy of all item by item judgements.	• There was a significant interaction effect between accuracy and the aware group. The unaware group made higher FOK ratings for incorrect responses than did the aware group. There was no difference in FOK ratings between groups for correct responses. The analysis of association between awareness, FOK ratings, memory accuracy varied across task condition showed a main effect of accuracy - the FOK ratings varied depending on memory accuracy. There was no main effect of group or task condition.
2. Cosentino et al. 2015	14 - AD moderate 20 - HE	• Metamemory test: • Digit span; • PVLT • Biber figure learning test • GPG • MRI	• AD - one condition: standard. • HE - three conditions - standard, query or feedback. • Standard - Participants were asked to make prospective judgments for each item in response to written questions. • Query - identical to the standard, with the additional component of a retrospective evaluation after each item. • Feedback - identical to the standard except that the examiner provides feedback regarding memory performance after each item.	• Performance on the metamemory test was not significantly associated with education. Metamemory performance was associated selectively with right insular volume. The left insula has also been implicated in supporting aspects of self- awareness and we sought to determine the robustness of the apparent right lateralization.
3. Cosentino et al., 2011	42 - mild AD50 - HE	• MMSE • PVLT • ACED • Clinical ratings of awareness • Modified FOK task	• The examiner read about five people (their name and something about their background). After the first learning trial, predictions for memory performance were acquired one at time for each item. The participants had to select the correct answer in eight possible -The answer choices included the correct response, the correct answers for the remaining 4 stimuli, and 3 new distractors. • Feedback condition - the examiner told the participant if their answer was correct or incorrect prior moving onto the next item.	• There was no difference in metamemory score as a function of task condition. There was no difference in metamemory as a function of stimuli set in HE or AD group. Participants of both groups demonstrated no variability in their prediction. Awareness was particularly important for decision making capacity. Decision making-capacity related to medication management was diminished in the unaware group.
4. Souchay et al., 2002	16 - AD 16 - HE 16 - YA	• MMSE • WCST • FAS • Stroop test • Logical memory test • Buschke selective reminding test • FOK accuracy Index	• The subjects were asked to recall the target that corresponded to a given cue. They had to underline the words seen earlier. The FOK judgment response was either a yes or no. After making the FOK predictions, the recognition task was administered.	• FOK_- no differences were found in the proportion of yes and no judgment in the three groups. • FOK accuracy - The AD patients made significantly fewer hits and more misses for yes judgment than older subjects. Prediction errors were more frequent for yes judgments than for no judgments. AD patients tended to overestimate their performance. No significant difference was found between younger and older adults.
5. Correa et al., 1996	20 - AD 18 - MCI 18 - HE	• Standard neuropsychological test battery • MIA Questionnaire • BCRP • RAVLT • BVRT • Word and animal fluency tests	• Estimation of performance accuracy following the administration of selected memory test (i.e. postdictions of number of previously recalled words/designs). Rey Auditory Verbal Learning Test.	• AD Patients tended to overestimated their performance on the BCRP and RAVLT, while memory-impaired and control subjects showed a slight tendency to underestimated their performance. The discrepancy between postdiction and actual scores on the BCRP and RAVLT across groups was not significantly correlated with delayed recall, self-report memory change, informant report of memory change, the discrepancy between self- and informant report of memory change, intrusions or the proportion of correct intrusions. The AD subjects were not fully aware of their memory deficits.
6. Pappas et al., 1992	20 - AD 12 - HE	• KnowMemFOK test EpiMem FOK test	• KnowMemFOK test - The subjects were instructed to rate their confidence that their answer was correct using a 6-category scale. EpiMem FOK test - the subjects read each incomplete sentence for which they had failed to recall the correct answer. They were asked to indicate their FOK and, immediately thereafter, the sentence and 6 alternatives ending were read with the subject asked to select one.	• KnowMemFOK test - The AD patients answered significantly fewer questions than did the controls. The patients and controls did not differ in their percentage of correct recognitions bat they differ in their FOK. • EpiMem FOK test - The AD patients were profoundly impaired on the recall test. Neither the controls nor the AD group were able to predict their recognition performance.

AD: Alzheimer's disease; HE: health eldery; YA: young adult; MCI: mild cognitive impairment; PVLT: Philadelphia repeatable verbal learning test; GPG: graphic pattern generation; MRI: magnetic resonance imaging; MMSE: mini mental state examination; ACED: assessment of capacity for everyday decision making; WCST: Wisconsin card sorting test; FAS: verbal fuency; MIA: metamemory in adulthood; BCRP: the Buschke cued recall procedure; RAVLT: the Rey auditory verbal learning test; BVRT: the Benton visual retention test - multiple choice.

**Table 2:** Summary of studys that used Judgment of Confidence (JOC).

Study	Sample	Measures	Tasks	Main results
1. Szajer & Murphy, 2013	143 - early to moderate AD 143 - HE	• Odor Recognition - Memory Task • Odor Threshold	• The participant was presented a sequence of odor. For each item participant were instructed to respond "yes" if the stimulus had been previously presented during the familiarity phase, and "no" if it had not.	• The control group performed significantly better confidence interval on episodic recognition memory task than did the AD group, however, both groups reported levels of confidence that failed to accurately differentiate between correct and incorrect responses. • Education level emerged as a significant predictor of confidence levels for incorrect responses and false alarms. No significant effect of diagnosis was found.
2. Gallo et al., 2012	18 - mild AD 18 - HE	• Metamemory task • AQD	• Participants studied object words and correspondent pictures presented as colored photos or as line drawings, and then took a picture recollection test followed by a confidence judgment.	• Reduced accuracy in AD participants compared to controls • AD participants were less likely to make high-confidence responses than were controls • The average for correct responses was greater than incorrect response in all groups. Calibration error scores were significantly greater in the AD group compared to control group • AD participants demonstrated some accuracy • AD insight into their cognitive decline, as their self-ratings of everyday problems were greater than the self-ratings problems reported by controls. • Participants with mild AD were able to use confidence judgments to track the accuracy of their responses on a recollection test.
3. Moulin et al., 2003	16 - AD 16 - HE	• CANDEX assessment tool • MMSE • Family interview • Laboratory screening	• Participants need select the word that they had seen before from the new word. This could be done either visually (by pointing) or verbally. • After selecting the word, they thought to be a target in each pair they were presented and rate how confident they were that they had selected the correct answer from the pair.	• AD group memory performance was worse than control group. • AD group is less confident in their memory performance than control group. • Both groups are accurate at assigning confidence to their recognition performance and the lack of a group difference suggests that the AD participants are accurate as controls in assigning confidence to their recognition performance.

AD: Alzheimer's disease; HE: health eldery; MMSE: mimi mental state examination; AQD: anosognosia questionnaire-dementia; CAMDEX: Cambridge mental disorders of the elderly examination.

**Table 3:** Summary of studys that used Judgment od Learning (JOL).

Study	Sample	Measures	Tasks	Main results
1.Genon et al., 2016	17 - HE 23 - mild probable AD	• Memory task • MRI	• A subjective prediction/judgment about face-name memory task with unfamous people (episodic items) and famous people (semantic items). The participant had to choose among four levels to indicate his/her primary subjective judgment.	• Memory performance - the AD participants had significantly lower recognition performance than HE for episodic items but had a similar recognition performance for semantic items. • Subjective judgment for hits - In AD group, predictions low hits was significantly higher than predictions high hits. • Correlation between episodic memory measures and gry matter density in AD - The brain regions most reably identified inclued the right medial temporal lobe (with both hippocampus and parahippocampal córtex), posterior cingulate córtex (PCC) and ventrolateral pré-frontal córtex (VLPFC).
2.Thomas et al., 2013	Experiment 1: 27 - YA, 31 - HE, 24 - Very Mild AD Experiment 2: 26 - YA, 58 - HE, 32 - Very Mild AD Experiment 3: 27 - YA, 31 - HE, 24 - Very Mild AD	• Three levels of associated strength cue-word: unrelated, weakly associated, and strongly associated	• Experiment 1: JOL was made immediately followed each pair. Participants had 8s in which to make a response. • Experiment 2: JOL were made immediately after the cue-target presentation; therefore both the cue and target were likely available to participants during a given prediction. For participants to accurately predict memory performance, they are required to discount what they know during study and adopt the perspective of the examinee. • Experiment 3: One third consisted of a category cues paired with exemplars of the category (e.g., "vegetable-carrot"). One third of the pairs consisted of words that rhymed (e.g., hurt-dirt), and one third consisted of the first two letters of the target as the cue (e.g., ba-balloon).	• Intact metamemory monitoring processes in both older adults and people with DAT. Within the context of an episodic JOL task, both groups of participants demonstrated above chance prediction accuracy. • Neither normal nor pathological aging impaired the extraction and use of intrinsic cues on JOL. • However, the results of the first two experiments do suggest that the effective use of intrinsic cues changes as function of both normal and pathological aging. • All groups of participants demonstrated changes in performance as a function of extrinsic cues. However, younger and nondemented older adults demonstrated a steeper rise in performance between the letter and rhyming cue conditions as compared with people with AD.
3. Clare et al., 2010	236 - HE 67 - AD 13 - mixed dementia (AD and vascular)	• MMSE • MARS • RBMT • RBMT-E • MFS • MPS • The National Adult Reading Test • The Hospital • Anxiety and Depression Scale • The Positive Affect Index	• MFS or MFS-S - The participant is asked to judge how often she/he would be able to achieve this • MARS, MFS-S, MFS-I and MPS made as each sub-test of the RBMT or RBMT-E. • MFD - compering self- and informant rating by means of a corrected discrepancy score. • MPR - comparing postdiction rating (MPS) and objective test (RBMT) by means of a ratio score.	• -AD made significantly lower self-ratings of memory functioning and performance, and had significantly lower informant ratings of memory functioning, than controls. • -AD participants also differed significantly from controls on the memory functioning (MFD) and memory performance (MPR) discrepancy indices, reflecting greater discrepancies between self- and informant ratings and between postdiction ratings and objective test score. • Between one-half and two-thirds of participants with AD overestimate compared to either informant ratings or objective test score to a degree that is extremely rare in control group. A much smaller proportion of AD participants underestimate.

AD: Alzheimer's disease; HE: health eldery; YA: young adult; FTD: frontotemporal dementia; PVLT: Philadelphia repeatable verbal learning test; GPG: graphic pattern generation; MRI: magnetic resonance imaging; MMSE: mini mental state examination; WCST: Wisconsin card sorting test; FAS: Verbal fuency test; MARS: the memory awareness rating scale; RBMT: Rivermead behavioral memory test; RBMT-E: Rivermead behavioral memory test - extended version; MFS: isomorphic memory functioning; MPS: Memory Performance Scale; WMS-R: logical memory subtest; FCSRT: free and cued select riminding test; TMT: the trail making test.

**Table 4:** Summary of studys that used Global Prediction and others metamemory's tasks.

Study	Sample	Metamemory'sassessment	Measures	Tasks	Main results
1. Rosen et al., 2014	12 - FTD 14 - AD 35 - HE	• FOK • EOL • JOL • Retrospective CR • Response to feedback	EOL, memory encoding, JOL • Free Recall • FOK and recognition memory • Retrospective Confidence ratings (item-by-item) • Response to feedback	• EOL, memory encoding, JOL - after learning the word list, show the first word in each pair and asked to remember the second word pair. After seeing two examples, asked to say how many pairs they would likely be able to recall - predict how many of the words they would recognize from among the choices. • Free Recall - participants were shown the first word in each pair, one at time, and asked to recall the associate for each. • FOK and recognition memory - participants were shown the first word in each pair, one at a time, and reminded the correct associate among them. • Retrospective Confidence Ratings (item-by-item) - participants were shown the first word in each pair, one a time, and asked to rate their confidence that they had chosen the correct associate. • Response to feedback - participants had to rate how many pairs they had recalled correctly. Immediately following this feedback, they were asked to make another recall prediction about if give a similar list pair how many pairs will be remembered.	• Recognition predictions were higher then recall predictions in all groups. • AD and FTD patients appropriately lowered their estimates based on task conditions and experience with the specific stimuli. • Recall prediction 2 was higher than actual in all groups. FTD patients rated themselves similar to controls. • Significant impairments in FOK accuracy in FTD and AD, the deficit appeared more severe in FTD. • FTD participants used moderately confident and not very confident less often and used just guessing more often than controls. AD participants used moderately confident, not very confident and just guessing more often than controls. • Controls distributed their responses fairly evenly across all four FOK ratings but used the just guessing rating less than the others. AD patients responses were skewed toward lower ratings. FTD patients responses were quite aberrant. Most of their responses fell into the highly confident or just guessing categories. • The relationship between retrospective confidence and accuracy was significantly different in FTD and AD compared with controls. • Recall prediction 3, was lower than all prior predictions in all three groups. However, FTD patients did not lower their prediction commensurate with their score.
2. Shaked et al., 2014	109 - Mild to moderate AD 50 - HE	• Global cognition	• MMSE • PVLT • Biber figure learning test • FAS • GPG	• Global metamemory judgments prior to and following the presentation of the individual items, as well as judgments for each individual item. • Immediately after the first learning trial, predictions for memory performance were acquired - eight possible answers • The tester did not give feedback and moved on the next item.	• Metamemory was associated with both nonverbal fluency as well as nonverbal memory. • The study failed to find a significant association between metamemory and letter fluency, a verbally based executive task. • Neuropsychological evidence has indicated the involvement of right prefrontal regions in both metamemory and nonverbal executive tasks.
3. Schmitter-Edgecombe & Seelye, 2011	20 - AD 20 - HE	• Global prediction • On line e Offline metamemory	• Medical interview CDR • The telephone interview of cognitive status-modified • Memory Functioning Questionnaire • RAVLT • MFQ	• A global performance prediction paradigm - predicted the number of word they would remember both prior to after completing a list -learning memory test (online assessment memory). • Self-ratings about their everyday memory failures (offline assessment memory).	• AD group demonstrated poorer episodic memory performance than controls and overestimated their episodic memory performance. The AD group was significantly less accurate than control group. • AD group overestimated their everyday memory abilities. AD group learned disproportionately fewer words across the five learning trials than HE. AD group was able to successfully modify their predictions based on task experience. The memory predict accuracy of the AD group did not differ from the control group at postexperience. The memory prediction of the AD group was significantly better postexperience than at preexperience. Patients with AD were able to monitor their performances and subsequently use this information to derive more accurate postexoerience performance expectations.
4. Galeone et al., 2010	25 - MCI 15 - mild DA 21 - HE	• OJ • Subjective rating of memory function	• Italian Interdisciplinary network on AD • MAC-Q • Frontal Assessment Batery • Subjective rating of memory function • Objective judgement task	• Six-items questionnaire (the subject had to rate, for every items, the presence of daily life memory failures on a likert scale rating from 0 to 4. Caregivers completed an informant version of the scale in order to obtain a discrepancy score (SRD). • Objective judgement task - three lists of 10 not-semantically related words comparable for length. • The participants had to predict how many words they would be able to recall (pre-study prediction). After, they studied the 10 words of the list, one at a time for 2s each, reading each word aloud. • Subjects were asked to predict again their performance (post-study prediction) and then recalled the words of the list. • After the recall, the subject and the examiner brietly commented the task but no explicit feedback on performance was given.	• The AD performing worse than the MCI and these, in turn, worse than the HE. • While for MCI and AD accuracy did not change across the three lists, the accuracy of the HE increased from list 1 to list 3. • The three groups were more accurate at the post-list judgement only for the first list. • The HE overestimated their performance at the beginning of the trial, but progressively revised their prediction so that, at the third list, this was virtually perfect. Both AD and MCI subjects consistently overestimated their performance across the three lists. • A consistent proportion of AD and MCI subjects had reduced awareness of their memory disturbances at the clinical interview.
5. Hannesdottir et al., 2007	92 - early or unspecified AD 92 - HE	• OJD • SRD	• The experimenter rating (OJD) • SRD scale • ERS • memory test for faces • RAVLT • digit span • trail making test • Language • Visuospational function • intrusional Erros	• Objective judgment discrepancy - the subject makes a judgment on his/her performance on the memory tests. Following each memory test item, the participant was asked to estimate the percentage/number if items successfully recalled from the memory test. • Subjective rating discrepancy - Contains questions pertaining to different aspects of memory ability in everyday life. Participants were required to rate their ability to perform certain memory related activities as well as to estimate the frequency with which they made errors. Subjective rating discrepancy to different aspects of memory. For the patients, the questionnaire was also administered to informants in third person. • The experimenter rating - The scale required the experimenter to rate the level of anosognosia for memory deficit. The participant reported difficulties. This scale was administered for patients only.	• All anosognosia assessment methods used in this study (the OJD, SRD and ERS) revealed a significant difference between AD patients, showing more anosognosia for their memory functioning than the comparison group. • OJD judgement might be more highly related to monitoring of ongoing memory performance in addition to self-efficacy beliefs and memory knowledge and the on-line process, which in turn may rely on frontal lobe functioning. • The SRD and ERS measures may pick up on the development of awareness, or otherwise, in relation to integrating information about efficacy in different settings over a long period of time. The SRD Global ratings scale was significantly correlated with the SRD memory scale, but this might be predicted because the scales use similar techniques. However, the ERS measure correlated with the SRD global rating scale, but not the SRD memory scale. The ERS measure and SRD global rating scale provide a more of an overview of anosognosic deficit in general than the more specific areas explored by the SRD memory scale.
6. Souchay et al., 2003	16 - AD 6 - FTD 16 - HE	• WMS-R • FCSRT • WCST • TMT • FAS • Global Prediction Accuracy	• Global memory prediction	• Participants wrote down the number of items they believed they would recall on the final paired-associate recall test (before study prediction).	• AD and FTD patients predicted recalling as many word as control subjects. For the predictions before and after study, the analysis did not reveal any significant difference between AD patients and control group. For FTD patients, there was no significant difference for the predictions made before or after study. A significant difference between AD and controls in prediction accuracy measures both before and after study, with AD patients predicting more than they recalled and control subjects recalling more than they predict. FDT and AD patients seemed to be less accurated than control subjects in predicting their memory performance. A significant group difference between AD and FTD was found only for after-study prediction accuracy score, with FTD patients predicting more than they recalled to a greater extent than AD patients.
7. Moulin et al., 2000	16 - AD 16 - HE	• JOL • Recall readiness	• MEEM • Family Interview • Laboratory screening • Liver and thyroid function • Calcium and syphilis • Medical examination • JOL instruction	• Participants had to estimate how many times a word had been seen. And were required to remember as many as items as possible. • Three phases to this experiment: presentation, recall and recognition.	• HE group were better than AD group. • Beneficit of repetition in both groups. • Dissociation in the AD group between judgments of learning and allocation of study time. • HE showed repetition effects for both study time and their explicit judgments of how well they have learned the items and this is not the case of AD group.
8. Moulin et al., 2000	16 - AD 16 - HE	• JOL • Ranking judgment	• MMSE • Family Interview • Laboratory screening • Medical examination • Neuropsychological examination	**Experiment 1** • Recall readiness task. • immediate free recall test at the end of the list, with an unlimited time to recall. • After presentation of 10 words, there was a short recall • verbal free recall of the items that they had studied.JOL task: • This was the same as the recall readiness task, except that each item appeared for only 2s and was then masked. • Participants were prompted to rate how likely it was that they would be able to recall the word later. • **Experiment 2** • Ranking judgment - two successive trials of the same test. • 1. Asked to rank the words in a different order (ranked the most easy to remember to most difficult) • 2. This order was reserved.	• Giving participants as long as they like to maximize their recall performance does not improve recall. • AD recall is much lower than controls. The older adults recall significantly more easy words than difficult words. The AD group gave JOLs that were as sensitive to objective difficulty as controls. AD group spends a lot longer studying the words than controls. Examining metamemory sensitivity to items differences using both JOL and recall readiness measures, reveals no evidence of a deficit in the AD group relative to non-diseased controls. Higher JOLs for words subsequently recalled. AD group were less discriminating in their JOL ratings. No clear benefit of extra study time in recall of items in either group. AD patients were as sensitive as older adults in the ratings (JOL) they gave to items. • Memory control and recall readiness - no significant differences between groups. • recall performance was not affected by whether items were ranked from difficult to easy words or vice versa, nor did this factor interact with any other. • The HE recalled significantly more items. • Participants were sensitive to the objective differences between words and make their ranking judgements accordingly. The AD was as sensitive as HE in ranking the words, and neither group is more or less sensitive in trial 1 compared to trial 2. • Participants recalled more of words they rank as easy than the words they rank as difficult.
9. Moulin et al., 2000	16 - AD 16 - HE	• Global Judgment of performance	• Recall readiness task • Judgment of learning task	**Experiment 1** • The word list comprised 5 easy and 5 difficult items. • List 1 - participants studied each word for an unlimited time. • List 2 - presentation time was fixed (2s per word) and participants had to give each word a rating of how likely they were to subsequently recall it. **Experiment 2** • 10 words, 4 list; • The word list comprised: Easy unrelated (E-U), difficult unrelated (D-U), easy related (E-R) and difficult related (D-R). • Two levels of relatedness: related in which all the items came from the same semantic category, and unrelated in which none of the items came from the same semantic category and unrelated in which none of items came from the same semantic category.	• The AD patients tend to overestimate their recall performance, but that they revise their predictions downward after encoding. • AD group was less accurate overall. Both groups were more accurate in the second list. • All groups make more accurate poststudy prediction than pre-study predictions. • Only AD group revises its predictions by a significant amount once they have been presented the list. The AD group monitoring its memory performance and revises its estimates of performance. • The HE group outperforming the AD group. • More words were recalled from the easy list. The HE group was sensitive to difficultly but the AD group not. • Participants recall significantly more words from the related lists, and the AD group did not benefit from relatedness to the same extent as the HE group. • The AD group did not differentiate between easy and difficult list in their predictions. There was a strong trend in the HE group to make higher predictions of performance for easy lists. • Both groups estimate that they will recall more words from the related lists and both are aware of the benefit of semantic relatedness to recall. • AD group poststudy predictions are not sensitive to item difficultly but are as sensitive to semantic relatedness as those of the HE group. • The AD group was more discrepant in their predictions than the HE group. • Participants were more accurate in their poststudy predictions.

AD: Alzheimer's disease; HE: health eldery; MCI: mild cognitive impairment; FTD: frontotemporal dementia; MMSE: mini mental state examination; FOK: feeling of knowing; EOL: ease of learning; retrospective CR: Retrospective confidence rating; JOL: judgment of learning; OJ: objective judgment; OJD: objective judgment discrepancy; RSD: subjective rating discrepancy; ERS: the experimenter rating scale.

### Tasks and related outcomes

**FOK** – FOK consists of three phases - recall, judgment and recognition - and investigates prospective monitoring at the time of retrieval.^11^ It is performed after the recall attempt and reflects the participants’ capacity to monitor their performance by generating and using feedback from their own memory performance. In FOK tasks, participants are asked to estimate the likelihood of the recognition of information which they have failed to recall, either from semantic memory^1^^,^ 16 or from recently learned episodic memory information.^17^

A total of six studies used the FOK task.^6^^,^ 13 ^,^ 18 ^-^ 21 Some differences were found in metamemory tasks between these studies. Cosentino et al.^6^^,^ 13 ^,^ 18 used fictitious personal histories and their backgrounds to measure metamemory. This task consisted of four trials with five items each, yielding a total of 20 metamemory items. In 2016,^13^ the objective of the study was to evaluate the accuracy of judgements in PwAD with preserved awareness of the disease and in unaware PwAD. In 2015^6^ and 2011,^18^ they investigated metamemory using moderate PwAD^6^ and MCI,^18^ compared with health elderly (HE). They concluded that there was a significant interaction effect between accuracy and awareness of memory. The unawareness was related to participants’ higher FOK ratings for incorrect responses. The aware group provided higher FOK ratings for correct responses compared to incorrect responses. There was no difference in FOK ratings between groups for correct responses. Awareness was particularly important for decision-making capacity. Another finding was the non-association between metamemory and education. Neuroimaging exams showed that metamemory performance was selectively associated with right insular volume while the left insula has also been implicated in supporting aspects of self-awareness.^6^ The limitations of these studies include that overall judgements of capacity were not made in a dichotomous fashion by expert raters; the results do not comment on whether awareness directly affects the rating of an individual as capable or incapable of making daily decisions about medication management; and the cognitive battery used was extremely limited.

Souchay et al.^20^ and Correa et al.^19^ used a memory word list as a FOK task. Both studies showed that PwAD tended to overestimate their performance compared to HE. In Souchay et al.,^20^ there was a word list and participants were asked to recall the target that corresponded to a given cue. They underlined the words seen earlier. The FOK judgment response was either a yes or no. After making the FOK predictions, the recognition task was administered. The study used PwAD, HE and young adults (YA). The PwAD made significantly fewer hits and more misses for yes judgment than HE. Prediction errors were more frequent for yes judgments than for no judgments. PwAD tended to overestimate their performance. There was no significant difference between the memory of YA and HE. These findings show that metamemory is not associated with aging. Moreover, episodic memory may be more important than executive function in explaining FOK inaccuracy. Correa et al.^19^ evaluated metamemory in PwAD, MCI and HE by estimating performance accuracy following the administration of a selected memory test. They concluded that PwAD tended to overestimate their performance, while memory-impaired and control subjects showed a slight tendency to underestimate their performance. The study showed that the diminished awareness of memory impairment and deficient self-monitoring abilities are restricted to PwAD. In addition, the discrepancy between postdiction and actual scores across groups was not significantly correlated with delayed recall, self-report memory change, informant report of memory change, discrepancy between self- and informant report of memory change, intrusions or the proportion of correct intrusions.

Pappas^21^ used short sentences and participants had to predict how likely they would answer correctly on a 6-category scale. PwAD made significantly fewer hits and more misses for yes judgment than HE. These errors were more frequent for yes judgments than for no judgments. The findings suggested that PwAD tended to overestimate their performance.

Five studies used HE as a control group.^6^^,^ 18 ^-^ 21 One study compared aware and unaware PwAD.^13^ Four studies manipulated the condition of the task to evaluate whether it could influence participant performance.^6^^,^ 13 ^,^ 18 ^,^ 21 These studies showed no effect of the condition of the task on the results of metamemory monitoring. Also, PwAD performance was worse than HE. Although several studies showed that the PwAD tended to overestimate their memory performance,^6^^,^ 13 ^,^ 18 ^,^ 19 ^,^ 21 Pappas^21^ indicated that neither the HE nor the PwAD were able to predict their recognition performance, but showed accuracy for their recall performance. The study^21^ tended to cluster both groups’ confidence ratings into a single category.^21^ Overall, PwAD were less confident about their answers (both correct or incorrect) than controls. In this analysis, only the data for subjects who provided ratings for both correct and incorrect items were included.

**JOL** – JOL tasks require subjects to, “online”, predict the likelihood of subsequently recalling information about recently studied items.^1^^,^ 11 ^,^ 15 ^,^ 22 Therefore, it is a self-prediction of prospective memory. JOL involves an inferential judgment based on the individual’s prior knowledge of variables that will influence his or her memory performance. When people have a subjective sense that new information has not been learned sufficiently for later retrieval, then they may decide to apply memory strategies. Superior JOL is associated with superior learning.^14^^,^ 22

Three studies used JOL tasks.^10^^,^ 15 ^,^ 23 The predictions were made after each individual item or each cue-target pair. All three studies had HE as a control group. One study had YA in the experiment.^15^ Genon^10^ used a face-name memory as a JOL task. The subjective prediction/judgment was made about the face-name memory task with non-famous people (episodic items) and famous people (semantic items). The participant had to choose among four levels to indicate his/her primary subjective judgment. During the encoding phase, 85 unknown faces were associated with full names, and the metamemory question was: “Could you recognize his/her full name?” The participants had to indicate the probability of recognition using a four-point scale. The study showed that PwAD had significantly lower recognition performance than HE for episodic items, but had a similar recognition performance for semantic items. In the AD group, low hit predictions were significantly higher than high hit predictions.

This type of stimulus (face-name) was also used in the study of Clare,^23^ in which the participant had to predict his/her ability to recall the person’s name learned when a photograph of this person was shown. This study showed that PwAD had significantly lower self-ratings of memory functioning and performance, indicating that memory was rated as less efficient. PwAD also differed significantly from controls on the memory functioning and memory performance discrepancy indices, reflecting greater discrepancies between self- and informant ratings and between postdiction ratings and objective test scores. However, about one-half and two-thirds of the PwAD overestimated compared to either informant ratings or objective test score to a degree that was extremely rare in control group. The finding confirms that significant overestimation is a frequent, although not universal, feature among PwAD, and that underestimation is also reliably observed, although to a much lesser extent.

Thomas et al.,^15^ used cue-targets-cue words pairs as stimulus for JOL tasks and manipulated the set of cues using three levels of associated strength cue-words: unrelated, weakly associated and strongly associated. The study also evaluated the influence of extrinsic and intrinsic cues on participant performance. The study reported that the effectiveness of use of intrinsic cues changes in both normal and pathological aging and demonstrated changes in performance of both groups as a function of extrinsic cues. In summary, the study showed that intact metamemory monitoring processes could be seen in both HE and PwAD.

**JOC** – JOC refers to retrospective judgments of confidence. These judgments are made after recall or recognition. JOC are thought to be based on the strength of the underlying memory trace, ease of retrieval, on heuristics applied to the specific study and test conditions, and on the subject’s own memory. In this sort of task, participants are asked to judge the accuracy of their answer.^25^

Three studies investigated the metamemory JOC task.^12^^,^ 26 ^,^ 27 JOC refers to retrospective judgments of confidence. Szajer et al.,^26^ used odor stimuli to investigate JOC. The study investigated the effect of education on retrospective metamemory accuracy of odor recognition. The olfactory stimuli included 15 common household odors presented in amber colored glass jars. Odor stimuli were embedded in a context of visual stimuli. The odors were randomly selected and presented one at a time, embedded in the sequence of odor, face, symbol. The results showed that the control group performed significantly better on episodic recognition memory task than did the PwAD group. However, both groups reported levels of confidence that failed to accurately differentiate between correct and incorrect responses, showing that there was no significant effect of education on odor recognition accuracy. Education level emerged as a significant predictor of confidence levels for incorrect responses and false alarms. A limitation was that the study did not compare the olfactory task with another modality. Olfactory memory processing involves different regions than other modalities of memory processing.

One study^12^ used common object words and corresponding pictures. Pictures were presented as colored photos or line drawings. The participants studied these objects and then took a picture recollection test followed by a confidence judgment. The results showed that there was reduced accuracy in PwAD compared to HE. PwAD were less likely to make high-confidence responses than were controls. Calibration error scores were significantly greater in the PwAD group compared to the control group, but, despite this, PwAD demonstrated some accuracy. PwAD insight into their cognitive decline and their self-ratings of everyday problems were greater than the self-ratings of problems reported by HE. Mild PwAD were able to use confidence judgments to track the accuracy of their responses on a recollection test.

The third study employing the JOC task used a list of words.^27^ Participants selected a word that they had seen before from a new pair of words. This could be done either visually or verbally. After their response, they had to select a target in each pair they had been presented before and rate how confident they were that they had selected the correct answer. The results showed that AD group memory performance was worse than the control group and that PwAD were less confident in their memory performance than the control group. Both groups were accurate at assigning confidence to their recognition performance and the lack of a group difference suggests that the PwAD were as accurate as controls in assigning confidence to their recognition performance.

The three studies showed that the AD group was less confident than controls, but despite this, PwAD demonstrated some accuracy. Interestingly, Szajer et al.,^26^ showed that education level was a greater determinant than diagnosis, and emerged as a significant predictor for incorrect responses and false alarms.

### Global prediction metamemory tasks and other tasks

Global prediction or postdiction accuracy implies predictions about the amount of information that will later be recalled both prior to and after experience with a task.^14^

Nine studies used the global prediction metamemory tasks or mixed metamemory tasks to evaluated participant performance.^9^^,^ 14 ^,^ 24 ^,^ 28 ^-^ 33 Global prediction (or postdiction accuracy) refers to predictions about the amount of information that will later be recalled both prior to and after experience with a task.^14^

Rosen,^9^ investigated metamemory in FTD and compared with AD using global judgment, item-by item feeling of knowing and retrospective confidence rating (CR). The global judgment task entailed 20 word pairs presented consecutively on a computer and simultaneously read aloud by the experimenter. Participants had to say how many pairs they thought they would recall, followed by a second recognition prediction. In the feeling-of-knowing task, the participants were shown the first word in each pair, one at a time, and reminded that they would be shown a list of eight choices with the correct match among them. They were asked to estimate their likelihood of correctly recognizing its pair from the choice of eight. For CR, participants were again shown the first word in each pair, one at a time, and asked to rate their confidence that they had chosen the correct match from the eight choices. The results showed that recognition predictions were higher than recall predictions in all groups. AD and FTD participants appropriately lowered their ratings based on task conditions and experience with the specific stimuli. FTD participants rated themselves similar to controls. Significant impairments in FOK accuracy were found in FTD and AD. Controls distributed their responses fairly evenly across all four FOK ratings, but used the just guessing rating less than the others. In addition, PwAD responses were skewed toward lower ratings while FTD participants’ responses were quite aberrant. Most of their responses fell into the highly confident or just guessing categories. The findings showed that the relationship between retrospective confidence and accuracy differed significantly in FTD and AD compared with controls.

Shaked^28^ studied the relatedness of objective metamemory performance to cognitive tasks grouped by domain (executive function or memory), as well as by preferential hemispheric reliance defined by task modality (verbal and non-verbal). The tasks used were: Global cognition and premorbid IQ – MMSE and Wechsler Test of Adult Reading (WTAR). The results showed that metamemory was associated with both verbal and nonverbal memory, but the study failed to find a significant association between metamemory and letter fluency, a verbally-based executive task. Neuropsychological evidence has indicated the involvement of right prefrontal regions in both metamemory and nonverbal executive tasks. However, the study had a relatively limited battery of neuropsychological testing, particularly in the domain of executive functioning.

Schmitter-Edgecombe^14^ used the following paradigms: Global prediction and Online and Offline metamemory. The tasks used were the prediction of the number of words that would be remembered both prior to and after completing a list – learning memory test (online memory assessment) and Self-ratings about their everyday memory failures (offline memory assessment). The study^14^ showed that the AD group had poorer episodic memory performance than controls and overestimated their performance. The AD group was significantly less accurate than the control group and overestimated their everyday memory abilities. The AD group learned disproportionately fewer words across the five learning trials than HE. Despite this, the AD group was able to successfully modify their predictions based on task experience and the memory prediction accuracy did not differ from the control group at postexperience.

Galeone^29^ evaluated OJD and Subjective Rating of memory function. The tasks were: a 6-item questionnaire, in which the subject had to rate the presence of daily life memory failures on a Likert scale and caregivers completed an informant version of the scale to obtain a discrepancy score (SRD). The objective judgement task was assessed with three lists of 10 non-semantically related words comparable for length. Subjects had to predict how many words they would be able to recall (pre-study prediction). Subsequently, they studied the 10 words of the list, reading out each word aloud. Subjects were asked to predict their performance again (post-study prediction) and then recalled the words of the list. After the recall, the subject and the examiner briefly commented on the task, but no explicit feedback on performance was given. The study showed that AD subjects´ performance was worse than MCI subjects who, in turn, performed worse than the HE. The HE overestimated their performance at the beginning of the trial, but progressively revised their prediction so that, by the third list, prediction was virtually perfect. Both AD and MCI subjects consistently overestimated their performance across the three lists. A consistent proportion of AD and MCI subjects had reduced awareness of their memory disturbances at the clinical interview.

Hannesdottir^30^ used the OJD, SRD and ERS to measure metamemory. The OJD task was: each subject was asked to make a judgment on his/her performance on the memory test. Following each memory test item, the participants were asked to estimate the number of items successfully recalled from the memory test. Average OJD scores were computed separately for verbal and visual memory measures. The SRD task asked the participants to rate, on a five-point scale, their ability to perform certain memory-related activities as well as to estimate. The questionnaire was also administered to informants in the third person. On the ERS, the experimenter rated the level of anosognosia for memory deficit. All metamemory assessment methods used in the study (the OJD, SRD and ERS) revealed that PwAD showed more anosognosia for their memory functioning than the comparison group. OJD might be more highly related to monitoring of ongoing memory performance in addition to self-efficacy beliefs and memory knowledge and the on-line process, which in turn may rely on frontal lobe functioning. The SRD and ERS measures may detect the development of awareness, or otherwise, in relation to integrating information about efficacy in different settings over a long period of time. The SRD Global ratings scale was significantly correlated with the SRD memory scale, but this might be expected because the scales use similar techniques. However, the ERS measure correlated with the SRD global rating scale, but not with the SRD memory scale. The ERS measure and SRD global rating scale provide more of an overview of anosognosic deficit in general than the more specific areas explored by the SRD memory scale.

In Souchay,^24^ participants wrote down the number of items they believed they would recall on the final paired-associate recall test (before study prediction). The stimulus of this task was a word list with 20 critical cue-target words. The results suggested that AD and FTD participants predicted recall of as many words as control subjects. For the predictions before and after study, the analysis revealed no significant difference between PwAD and HE. For FTD participants, there was no significant difference for the predictions made before or after study. A significant difference between AD and HE in prediction accuracy measures both before and after study, with PwAD predicting more than they recalled and control subjects recalling more than they predicted. FDT and AD participants seemed to be less accurate than control subjects in predicting their memory performance. A significant group difference between AD and FTD was found only for after-study prediction accuracy score, with FTD participants predicting more than they recalled to a greater extent than PwAD.

Moulin^31^^-^ 33 assessed global prediction and JOL using a word list. The tasks used were to estimate how many times a word had been seen, to rate how likely they would be able to recall the word later and to rank the words in a different order (ranked from the easiest to remember to the most difficult and vice versa). The results showed that the HE group performed better than the AD group. There was a dissociation in the AD group between judgments of learning and allocation of study time. The HE group showed repetition effects for both study time and their explicit judgments of how well they had learned the items (this was not observed in the AD group). The AD group showed on the JOL that they were as sensitive to objective difficulty as controls, but spent a lot longer studying the words than controls. Examining metamemory sensitivity to item differences using both JOL and recall readiness measures, revealed no evidence of a deficit in the AD group compared to non-diseased controls, but the AD group were less discriminating in their JOL ratings.^31^^-^ 33 Participants were sensitive to the objective differences between words (easy or difficult) and made their ranking judgements accordingly. Participants recalled more of the words they ranked as easy than the words they ranked as difficult. Despite the tendency of the AD group to overestimate their recall performance, they revised their predictions downward after encoding. Interestingly, all groups made more accurate post-study predictions than pre-study predictions.

## DISCUSSION

The aim of this study was to identify the types of tasks used to evaluate metamemory monitoring, the stimuli used in these tasks, their limitations and the outcomes in PwAD.

Different tasks and stimuli were used to evaluate metamemory monitoring in PwAD. The most common tasks used were the JOL, FOK, JOC and Global Prediction. Some other assessments had the same definition as these four classifications. For example, online assessment of memory was used in the same context as Global Prediction, Objective Judgement as the same as the JOL, and CR the same as the JOC. This variation in terms hinders better categorization of metamemory tasks, homogeneity of concepts and methodological consistency.

SRD or SR are important tasks that involve discrepancy between PwAD and caregivers’ judgement of memory.^30^ These kinds of task are important to gain a better understanding of PwAD performance, because the discrepancy shows how metamemory judgement impacts caregivers. The discrepancies are commonly used to evaluate awareness of disease in PwAD,^34^ but, in terms of metamemory monitoring, further research is still needed.

The most commonly used stimulus was the word list. The number of words in the list varied between 10 and 20 and the most common test used was the RAVLT.^14^^,^ 19 ^,^ 30 These types of stimuli were used in all metamemory monitoring tasks i.e. the JOC, FOK, JOC and Global Prediction.

One study^26^ used odor stimuli to access metamemory monitoring. Stimuli that involve other sensory organs are extremely important to understand the complexity of metamemory monitoring and their impact on daily life. However, there were no comparative studies allowing us to generalize these results, as no studies have focused on other sensory organs, such as hearing. It is very important to invest in research focused on different sensory organs, as it enables other kinds of metamemory assessment in AD.

The articles also showed that PwAD usually had better monitoring for FOK^31^^-^ 33 and JOC tasks than for the JOL, regardless of the stimuli used.^14^^,^ 32 It is important to emphasize that the accuracy after encoding is better among cognitively healthy people, because they can revise their estimates. A study^32^ suggested that PwAD can update their memory predictions through their own spontaneous feedback about their individual performance. Thus, PwAD have similar results compared to healthy people on the FOK task. Taking this point into consideration, we may assume that repeated exposure may enable PwAD to make more accurate predictions of performance. It seems that any apparent deficit in global awareness among PwAD is a result of the memory deficit.^32^ However, there were no information on whether the increase in memory awareness through repeated testing in the AD group was converted into an enduring recall.^32^ When the tasks were analyzed in the same metamemory monitoring, it was observed that for pair target-cue words, there was fluctuation in monitoring ability according to cue type. Cues that did not have associative strength with the target word were associated with significantly worse recall.^15^ This point is interesting because intrinsic cues are useful, as they are easily extracted from to-be-remembered stimuli, and participants can use *a priori* knowledge developed through years of experience to generate predictions of memory ability.^15^

In contrast, recall may be better for highly related word pairs.^15^ Thomas et al.^15^ showed that, although younger adults demonstrated dramatic increases in cued recall performance for highly associated word pairs, PwAD also demonstrated improvement in cued recall as associative strength increased, but the increase was not as great as that found in younger adults. This study was the first to examine resolution in these groups with this kind of extrinsic manipulation and, therefore, could not posit an explanation for these findings until they have been replicated in future studies. Thus, it may be affirmed that extrinsic cues had a modest effect on average JOLs.

The capacity of PwAD to modify their predictions was associated with task experience and this is central for future studies and interventions. Thus, regarding metamemory monitoring, a study including Global Prediction tasks may be the most effective to capture the complexity of the metamemory concept. In addition, the predictions in metamemory are more inaccurate than the retrospective judgement about cognitive performance.^14^^,^ 32 For Schimitter-Edgecombe and Seelye,^14^ PwAD were able to successfully self-monitor their memory abilities, updating memory knowledge based on task experience. While the AD group predicted that their delayed recall for the word list would be at a similar level to controls preexperience, the postexperience of PwAD predicted a significantly poorer level of recall than controls. Therefore, the memory prediction accuracy of the PwAD did not differ from that of the control group at postexperience. This pattern of prediction upgrading is thought to reflect online monitoring and suggests that PwAD can monitor their performances and subsequently use this information to derive more accurate postexperience performance expectations.^14^ The limitations of this study included the small sample size and the fact that the participants were a group of well-educated, Caucasian older adults, precluding the generalizing of these results to other populations of PwAD.

Moulin et al.,^32^ showed that the AD group was not sensitive to item difficulty between lists, whereas the control group comprised of healthy elderly was. In addition, PwAD recall did not vary significantly across list types. Thus, it is assumed that the AD group was correct to be insensitive in their predictions, because the relationship between their subjective predictions and their actual recall was wholly appropriate.^32^ This finding may be evidence that metamemory monitoring may be intact in PwAD. PwAD use information gained while processing the to-be-remembered items to revise their predictions of subsequent performance, showing that they are sensitive to factors operating during encoding.^32^

We found that few studies associate objective metamemory tasks with neuroimage findings. Further investigation about the relationship between brain structure and objective metamemory tasks is necessary.^6^^,^ 10 In addition, only one study^18^ focused on the impact on metamemory functioning in daily life care.^18^ Future research should longitudinally investigate the patterns of change of metamemory impairment and deficits in functional capacity.

The present study has some limitations. We did not assess the risk of biases within and across the studies. Also, we did not register the review on the PROSPERO database. Despite these limitations, our findings may represent a significant contribution to the area of metamemory in AD, since there are few systematic reviews on the subject.

In conclusion, the study has shown that PwAD have deficits in metamemory tasks and usually overestimate their function and performance. When the judgment is made in forward-looking vision, as occurs for the JOL and JOC, the loss in metamemory is higher because the PwAD cannot use cognitive features like updating their expectations across repeated list exposure.^32^ The metamemory FOK task, however, indicated that PwAD can maintain the judgments of their metamemory. If PwAD can monitor memory, even in some gross manner, it is reasonable to try to improve their control of memory with behavioral interventions. Additionally, it is reasonable to reject catastrophic failure in metamemory monitoring as a contributory factor to the poor episodic memory performance of PwAD.

This type of research can help caregivers and clinicians conduct effective interventions to engage PwAD in treatment and care. Clinically, our observations can be used to improve cognitive interventions by helping the best choice of tasks and their potentials and limitations. In addition, the knowledge on the patterns of change of metamemory in AD can help clinicians and caregivers to develop interventions aimed at its preservation, as well as to identify compensatory styles of functioning. Also, our study may aid further research to make projections of future paradigms for assessment of metamemory in AD, which in turn may help improve the well-being of PwAD and their caregivers. Thus, this systematic review provides initial evidence for the use of metamemory measures as part of a broader assessment when evaluating the presence of AD.
